# Development of a Large-Volume Concentration Method to Recover Infectious Avian Influenza Virus from the Aquatic Environment

**DOI:** 10.3390/v16121898

**Published:** 2024-12-10

**Authors:** Laura E. Hubbard, Erin A. Stelzer, Rebecca L. Poulson, Dana W. Kolpin, Christine M. Szablewski, Carrie E. Givens

**Affiliations:** 1 U.S. Geological Survey, Upper Midwest Water Science Center, 1 Gifford Pinchot Drive, Madison, WI 53726, USA; 2 U.S. Geological Survey, Ohio-Kentucky-Indiana Water Science Center, 6460 Busch Blvd, Ste 100, Columbus, OH 43229, USA; eastelzer@usgs.gov; 3 Southeastern Cooperative Wildlife Disease Study, Department of Population Health, College of Veterinary Medicine, University of Georgia, 589 D.W. Brooks Drive, Athens, GA 30602, USA; rpoulson@uga.edu; 4 U.S. Geological Survey, Central Midwest Water Science Center, 400 S. Clinton St., Rm 269, Iowa City, IA 52240, USA; dwkolpin@usgs.gov; 5 Centers for Disease Control and Prevention, Influenza Division, Atlanta, GA 30329, USA; lqz9@cdc.gov; 6 U.S. Geological Survey, Upper Midwest Water Science Center, 5840 Enterprise Drive, Lansing, MI 48911, USA; cgivens@usgs.gov

**Keywords:** avian influenza, virus, infectious, method, water, environment

## Abstract

Since late 2021, outbreaks of highly pathogenic avian influenza virus have caused a record number of mortalities in wild birds, domestic poultry, and mammals in North America. Wetlands are plausible environmental reservoirs of avian influenza virus; however, the transmission and persistence of the virus in the aquatic environment are poorly understood. To explore environmental contamination with the avian influenza virus, a large-volume concentration method for detecting infectious avian influenza virus in waterbodies was developed. A variety of filtering, elution, and concentration methods were explored, in addition to testing filtering speeds using artificially amended 20 L water matrices (deionized water with sterile dust, autoclaved wetland water, and wetland water). The optimal protocol was dead-end ultrafiltration coupled with salt solution elution and centrifugation concentration. Using this method, infectious virus was recovered at 1 × 10^−1^ 50% egg infectious dose per milliliter (EID_50_/mL), whereas viral RNA was detected inconsistently down to 1 × 10^0^ EID_50_/mL. This method will aid in furthering our understanding of the avian influenza virus in the environment and may be applicable to the environmental detection of other enveloped viruses.

## 1. Introduction

Outbreaks of highly pathogenic avian influenza (HPAI) virus A/goose/Guangdong/1/1996 (Gs/GD) clade 2.3.4.4 lineage H5 have caused unprecedented numbers of mortalities in wild birds, domestic poultry, and various terrestrial and marine mammals across Europe, Asia, Africa, the Americas, and most recently Antarctica [1,2]. Wetlands are plausible environmental reservoirs of avian influenza virus (AIV) [3,4,5]. The fecal shedding of AIV from wild birds into surface water can lead to transmission among waterfowl primarily via the fecal–oral route [6,7,8,9,10]. Previous studies have suggested that AIV, including the current A(H5Nx) subtypes, may remain infectious for long periods in surface water; however, the persistence of AIV in the environment is poorly understood [11,12]. The role and contribution of the aquatic environment in the transmission of AIV among wild birds and domestic poultry is a documented, critical research gap [10]. In addition, other species, including mammals and humans, using these water sources as habitats, drinking water, or for recreation could be exposed via primary or secondary contact.

Concentration methods are common for the detection of viruses from environmental waters due to their low concentrations and have been used extensively in previous applications, including SARS-CoV-2 [13,14,15]. Many techniques exist for the concentration of virus from water, including ultrafiltration; electronegative and electropositive filtration; flocculation/precipitation; and ultracentrifugation [16,17]. Flocculation/precipitation and electronegative/electropositive filtration rely on particle charge, whereas ultrafiltration relies on size exclusion. Many of the viruses previously tested were related to human gastroenteritis or respiratory illness, such as enteroviruses, noroviruses, and adenoviruses [18,19]. In addition, many of these concentration methods were developed for molecular (PCR) detection and do not examine infectious viruses. The detection of infectious viruses (i.e., potentially transmissible viruses) is important for environmental health surveillance.

To date, there is no widely accepted method to recover infectious AIV from water. Several studies have examined methods to determine the molecular presence of AIV in water using various filters or membranes, elution solutions, and concentration methods; however, few studies have extensively examined the recovery of viable viruses [3,4,20,21,22,23,24,25]. Past recoveries of infectious AIV have been minimal, from no recovery to approximately 1% from surface waters [3,4].

With continuing HPAI A(H5N1) detections throughout the Americas, incorporating the environmental sampling of infectious HPAI into current biosurveillance is necessary and may represent a complementary approach to sampling animals. The first step is to develop and validate a method to recover infectious AIV from the large-volume filtration of water.

## 2. Materials and Methods

### 2.1. Design of Tests 1 and 2

Two laboratory experiments were designed to answer specific questions before the final procedure was tested in a field study [26]. The use of personal protective equipment and other BSL2+ laboratory safety procedures were followed during all water collection and laboratory experiments [27].

**Figure 1:**
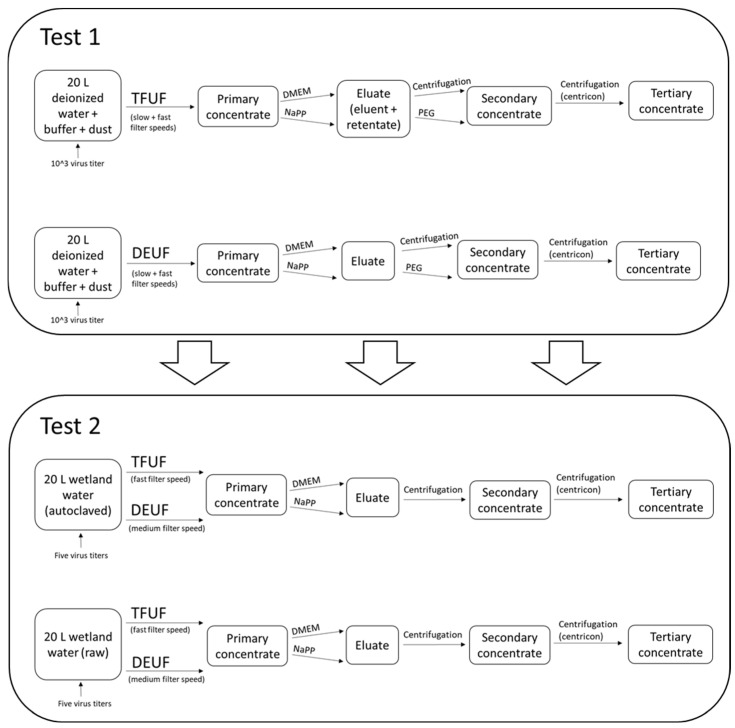
Schematic showing general steps in filtration and processing of samples in both Test 1 and Test 2. Test 1 results informed Test 2. TFUF, tangential flow ultrafiltration; DEUF, dead-end ultrafiltration; DMEM, Dulbecco’s Modified Eagle Medium; NaPP, sodium hexametaphosphate; PEG, polyethylene glycol precipitation.

Test 1 was designed to determine the optimal procedure(s) (a combination of pump speed, filtering type, elution solution, and secondary concentration) to recover infectious AIV from the large-volume filtration of environmental water (Figure 1, Table 1). Requirements to proceed in Test 2 included the recovery of infectious AIV and the molecular detection of AIV from the concentrated water sample. Considerations were also given to the consistency of elution solution source(s) and recovery variation among pump speeds. The purpose of Test 1 was to determine the pump speed(s), filtration type(s), and elution solution(s) in order to subsequently test with a natural matrix in Test 2 (i.e., wetland water). Test 2 was designed to determine the minimum concentration for the recovery of infectious AIV and whether biotic components affected the recovery of infectious AIV. Three treatments that met the requirements from Test 1 were then tested with natural wetland water in Test 2 (Figure 1, Table 1).

### 2.2. Avian Influenza Virus Propagation

A duck-origin low-pathogenicity (LP) AIV (A/mallard/Minnesota/UGAI14-2812/2014 (H3N8); hereafter, LP H3N8) was propagated by a second passage in 9–11-day-old embryonated chicken eggs (ECEs) at 37 °C, as previously described (UGA IACUC A2019 04-001-A3) [28]. The infectious titer of the viral stock was determined in ECEs after one −80 °C freeze–thaw to account for any viral loss due to this process. Viral titer was calculated using the method of Reed and Muench as 10^7.75^ 50% egg infectious doses per milliliter (EID_50_/mL) [29]. Briefly, 10-fold serial dilutions of concentrated water samples were performed in Dulbecco’s phosphate-buffered saline + antibiotics/antimycotics (penicillin G 200 units/mL, Streptomycin 200 µg/mL, and Amphotericin B 0.5 µg/mL; Sigma Aldrich, St. Louis, MO, USA; hereafter, 2× Abs) and then used to inoculate each of five 9–11-day-old ECEs at 0.1 mL per egg. Egg infectious dose 50 was calculated by Reed and Muench, based on hemagglutinating activity two to three days post-inoculation and confirmation of endpoint by rRT-PCR targeting the IAV matrix gene. The virus was aliquoted into single-use cryovials at 3 mL volume and stored at −80 °C until shipment, on dry ice, to the testing laboratory for utilization in the trial.

### 2.3. Water Matrix and Virus Information

Each 20 L of polypropylene carboy was sterilized before use with a 0.6% sodium hypochlorite solution followed by a 0.05% sodium thiosulfate solution and then rinsed three times with deionized (DI) water. The water matrices for the development and validation of the method were twofold; initially, a matrix of 20 L DI water with buffer (KH_2_PO_4_ + MgCl_2_; Table S1) [30] and 5 g of sterile dust (ISO 12103-1, A1 Ultrafine Test Dust; Powder Technology Inc., Arden Hills, MN, USA; Table S1) [30] was added to each 20 L carboy to test different filtration, elution, and concentration procedures that may yield infectious avian influenza virus (Test 1). To determine the final optimized method (Test 2), natural wetland waters were collected from a waterfowl refuge in Columbus, OH, USA (Table S2) [30]. This waterbody was selected for accessibility and as an approximate representation of migratory waterfowl wetlands in the midwestern United States. Samples were collected by immersing the 20 L carboys 6 to 12 inches below the water surface. The objective of our wetland water collection was to obtain consistent sampled water between carboys (i.e., no attempt to collect water that was representative of the entire water column). Thus, we sampled water at a depth of 12 inches to avoid both surface scum and bottom material. The week prior to Test 2, wetland water was collected, autoclaved at 121 °C for 90 min within 8 h of collection, and stored at room temperature at the U.S. Geological Survey (USGS) Ohio Water Microbiology Laboratory in Columbus, OH, USA [31]. Raw wetland water samples were collected the day before the experiment and transported at a maximum air temperature of 10.6 °C (Table S3) [30] along with the autoclaved carboys to the USGS Michigan Bacteriological Research Laboratory in Lansing, MI. Forty-seven of the 20 L carboys contained autoclaved wetland water (with biotic components removed), and ten of the 20 L carboys contained raw wetland water (with biotic components intact) (Table 1). The ten 20 L carboys that were not autoclaved were held at an average air temperature of 14.5 °C (13.2 to 15.3 °C; Table S4) [30] and processed within 27 h of collection to reduce the microbial degradation of the wetland water matrix.

For Test 1, each 20 L carboy was spiked with 1 × 10^3^ EID_50_/mL LP H3N8 with the exception of the three negative controls, which were filled with DI water, buffer, and test dust or DI water and buffer (Table 1 and Table S5). For Test 2, a dilution series was used to determine the minimum concentration of virus for the recovery of infectious AIV. The following quantities were tested: 1 × 10^3^, 1 × 10^2^, 1 × 10^1^, 1 × 10^0^, and 1 × 10^−1^ EID_50_/mL LP H3N8. After the virus was added to the 20 L carboy, the carboy contents were mixed on a magnetic stir plate for approximately five minutes to ensure the virus was evenly distributed. Test 2 had three negative controls (20 L carboys filled with DI water; Table S6) [30].

### 2.4. Filtering Methods

**Figure 2:**
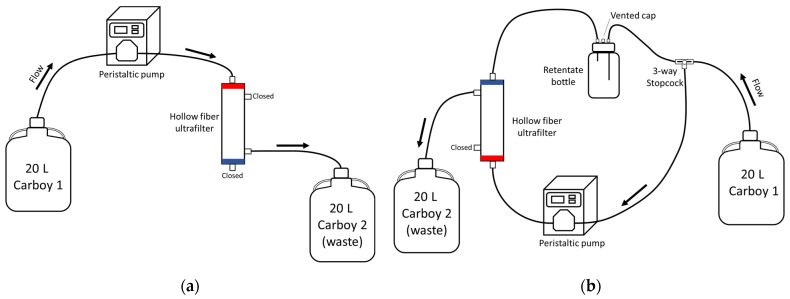
Schematic of setups for filtering water samples: dead-end ultrafiltration (**a**) and tangential flow ultrafiltration (**b**). Dead-end ultrafiltration setup from Smith and Hill [15] with modifications. Tangential flow ultrafiltration setup from Hill et al. [18] with modifications.

REXEED-25S (Asahi Kasei Kuraray Medical Co., Ltd., Tokyo, Japan) ultrafilters (UFs) with a polysulfone membrane and a 2.5 m^2^ filter area were used for Tests 1 and 2. Ultrafiltration relies on size exclusion, concentrating viruses from water samples by sieving rather than adsorption or sedimentation [32]. Two ultrafiltration methods were tested: dead-end ultrafiltration (DEUF) and tangential flow ultrafiltration (TFUF) [15,18]. During DEUF, the water is passed through the filter membrane, the solids are trapped in the filter, and the filtrate is released as waste (Figure 2a). With TFUF, the majority of the water flows tangentially across the surface of the filter rather than onto the filter, removing fluids while keeping the solute molecules (Figure 2b) [18].

Spiked (1 × 10^X^ EID_50_/mL) water was drawn from the 20 L carboy with a peristaltic pump (Masterflex, Radnor, PA, USA) using platinum-cured tubing (Masterflex I/P 26, L/S 36; VWR, Radnor, PA, USA) and pumped through the UF using either DEUF [15] or TFUF [18]. Following each ultrafiltration, tubing was autoclaved at 121 °C for 45 min and discarded as biological waste. Connectors were sterilized after each ultrafiltration by soaking them in a 0.6% sodium hypochlorite solution for 30 min followed by a 0.05% sodium thiosulfate solution for a minimum of 5 min and then rinsed three times with DI water. During Test 1, pump speeds of approximately 1.0 L/min (“fast”) and 0.20 L/min (“slow”) were tested. During Test 2, pump speeds of 1.0 L/min (TFUF; “fast”) and 0.50 L/min (DEUF; “medium”) were used (Table 1). Approximately 250 mL of sterile DI was used to rinse the carboy and pump the remaining water. The DEUF protocol and setup used were modified from those of Smith and Hill [15]. Modifications to the DEUF protocol included the exclusion of the pressure gauge, and the permeate (waste) was not analyzed. Before TFUF, UFs were blocked with 0.1% sodium hexametaphosphate (hereafter referred to as NaPP; Table S1) [30], and the filtration setup was modified from Hill et al. [18] (Figure 2b). TFUF modifications were the exclusion of a pressure gauge and syringe pump.

Following the development of this method, the distribution of the REXEED 25-S UF was discontinued in North America, and notification occurred in April 2023. A follow-up test was conducted comparing commercially available UFs in North America with a 2.5 m^2^ filter area: Elisio-25H (Nipro, Bridgewater, NJ, USA) and Optiflux F-250 (Fresenius Medical Care, Waltham, MA, USA). Due to limited available stock, a partial set of samples was analyzed using REXEED 25-S. Both filters (Elisio-25H, Optiflux F-250) performed similarly to the REXEED 25-S (Table S7) [30].

### 2.5. Elution Methods

During Test 1, the following elution solutions were tested: Dulbecco’s Modified Eagle Medium (DMEM; Corning 15017CV), NaPP, sterile DI, and a custom DMEM-filled elution can (Innovaprep LLC, Drexel, MI, USA; Table 1 and Table S1). Test 2 elution solutions included NaPP and DMEM (Table 1). Following each elution, UFs and tubing were autoclaved at 121 °C for 45 min and discarded as biological waste.

**Figure 3:**
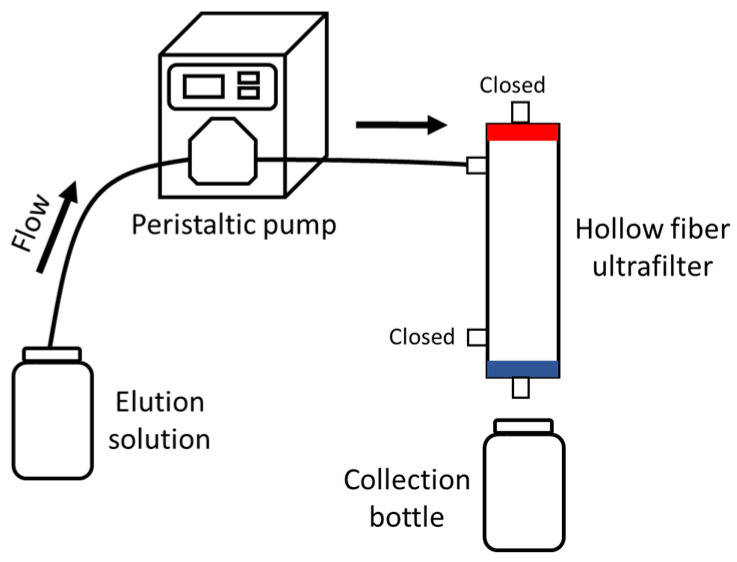
Schematic for elution of ultrafilters filtered using dead-end ultrafiltration from Smith and Hill [15] with modifications.

Elution solutions were drawn from either a clean and sterile graduated cylinder (NaPP, sterile DI) or directly from the container (DMEM) using a peristaltic pump (Masterflex, Radnor, PA, USA) with Masterflex platinum-cured tubing (types I/P 26, L/S 36) and pumped through the UF using either DEUF or TFUF. A sterile collection bottle was used to collect the eluent from DEUF (Figure 3). In TFUF, a two-step elution procedure was conducted: a forward rinse with sterile DI water was run through the filter followed by a backflush with sterile DI water to elute microbes that may have adhered to the filtration media.

### 2.6. Concentration Methods

For Test 1, LP H3N8 present in the eluate was further concentrated using modifications of polyethylene glycol precipitation (PEG) or solely through centrifugation. PEG has been used extensively for viral concentrations and the molecular detection of viral RNA and DNA [3,33,34,35], and more recently, PEG has been used to concentrate SARS-CoV-2 in wastewater [36,37,38,39]. During PEG precipitation, an 8% *w*/*v* PEG 8000 (Fisher BioReagents, Waltham, MA, USA) 0.2 M NaCl solution (Table S1) [30] was added to the eluate and placed on a magnetic stirrer at 4 °C for two hours [40]. Eluate was dispensed into 250 mL centrifuge tubes (Corning, Corning, NY, USA) and centrifuged at 4700× *g* for 45 min at 4 °C to pellet large particles. The supernatant was removed, and 1 mL of 0.15 M sodium phosphate was added to the tube to resuspend the pellet.

During centrifugation only, the eluate was transferred to 250 mL centrifuge tubes, centrifuged at 3500× *g* for 15 min at 4 °C, and the pellet was discarded. After either PEG or centrifugation, samples were transferred to a pre-rinsed (sterile DI) 30,000 d Centricon Plus-70 filter unit (EMD Millipore, Burlington, MA, USA) and centrifuged according to the manufacturer’s instructions, and as described previously described [18], to concentrate viral particles to a median volume of 899 µL (range, 332–2310 µL; Test 1) and 2300 µL (range, 1350–6060 µL; Test 2). Concentrated virus samples were stored at 4 °C and shipped overnight at 4 °C to the Southeastern Cooperative Wildlife Study (SCWDS; University of Georgia; Athens, GA, USA) for further processing.

### 2.7. Avian Influenza Virus Isolation, Confirmation, and Titration

Samples were stored at 4 °C upon receipt at the SCWDS. Prior to processing, 2× Abs were added to each, and samples were then vigorously vortexed and allowed to sit at room temperature for approximately 15 min. Nucleic acids from water samples were extracted using the MagMAX-96 AI/ND Viral RNA Isolation Kit (Ambion/Applied Biosystems, Foster City, CA, USA) on the KingFisher magnetic particle processer following a modified MagMAX-S protocol, as previously described [41], two to four days post-collection and screened by real-time reverse transcriptase polymerase chain reaction (rRT-PCR) targeting the AIV matrix gene where a cycle threshold (Ct) value < 40 was considered “positive” [41,42]. Inhibition assessment is not included in the published molecular methods frequently used in diagnostics of avian influenza virus and, therefore, was not included in Tests 1 and 2.

Virus isolation (VI) was performed in ECEs two to eight days post-collection. ECEs were monitored daily for viability, and after a 48 h incubation of the ECEs at 37 °C, amnioallantoic fluids were tested by a hemagglutination assay (HA) using 0.5% chicken red blood cells (RBCs) [43]. All samples that did not hemagglutinate RBCs after one passage were subjected to a second passage in ECEs, and procedures for the HA assay followed, as noted above. Resultant viral isolates were extracted using the Viral RNA Mini Kit (Qiagen, Germantown, MD, USA) as per the manufacturer’s instructions and further screened using rRT-PCR assays targeting the AIV matrix gene, as above.

Select filtered water samples from which infectious AIV was isolated were subjected to endpoint titration in ECEs [44]. Briefly, ten-fold serial dilutions of each AIV VI-positive water sample were prepared in 1× Dulbecco’s phosphate-buffered saline (Sigma Aldrich) supplemented with 2× Abs from 10^−1^ to 10^−6^. Five ECEs per dilution were each inoculated with 0.1 mL via the allantoic route. After 48 h of incubation at 37 °C, the HA assay was performed; viral RNA was extracted (Qiagen) from the HA-positive eggs at the endpoint dilution and screened in rRT-PCR to confirm the presence of AIV. Titer endpoints were calculated as described in [29].

### 2.8. Virus Recovery Efficiency and Statistics

Virus recovery was determined in two ways: (1) a subset of primary samples that yielded an AIV isolate was titrated in ECEs through a ten-fold serial dilution series until an endpoint was reached as described above; (2) a percent recovery was calculated from rRT-PCR. In this study, rRT-PCR-positive controls (known AIV matrix avian-origin isolates and avian-influenza-matrix-transcribed RNA) were not quantified and were used only as confirmations of a successful extraction and rRT-PCR. The detection of infectious virus (presence/absence) was the objective of Tests 1 and 2; however, a further avenue would be to explore the quantification or abundance of these infectious viral particles. Since concentrated water sample volumes were not consistent among the replicates in this data set, a standard curve was introduced to incorporate these different volumes in the recovery calculations. The standard curve was generated only to compare recoveries between tests in this particular data set. In order to calculate percent recoveries, a quantified seven-point standard curve from a later study was applied (Table S8) [30]. The percent virus recovery was calculated as follows:

(1) $$\frac{spiked\;sample\;concentration}{original\;virus\;seed\;concentration}\times \;100\;=\;percent\;recovery$$

The Kruskal–Wallis rank sum test was used to determine the statistical significance of differences in the matrix treatment, virus titer, filtering speed, filtering type, elution type, and concentration method. Dunn’s tests were performed for multiple pairwise comparisons on those Kruskal–Wallis results that were statistically different (i.e., *p* < 0.05).

## 3. Results

### 3.1. Test 1

The virus titer for the spike remained the same throughout Test 1, allowing us to assess recovery with the different techniques. LP H3N8 was isolated regardless of treatment (a combination of filtering type, filter speed, elution solution, and secondary concentration; Table S5) [30]. While there was no standard curve used in the rRT-PCR analysis, we can compare the recovery results with each other using an assay standard curve (Table S8) [30].

**Figure 4:**
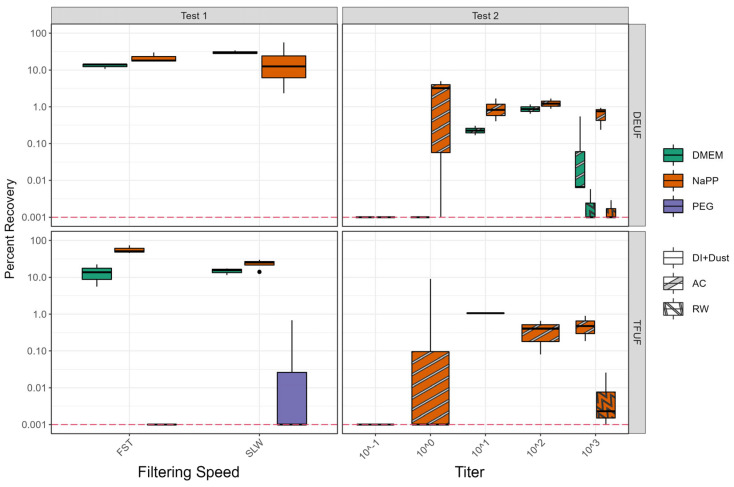
Percent recoveries calculated from rRT-PCR (real-time reverse transcriptase polymerase chain reaction) Ct (cycle threshold) values from Test 1 (deionized water + sterile dust) and Test 2 (autoclaved and raw wetland water). TFUF, tangential flow ultrafiltration; DEUF, dead-end ultrafiltration; FST, fast pump speed (1.0 L/min); SLW, slow pump speed (0.20 L/min); DMEM, Dulbecco’s Modified Eagle Medium; NaPP, sodium hexaphosphate; PEG, polyethylene glycol precipitation; DI+Dust, deionized water and sterile dust; AC, autoclaved wetland water; RW, raw wetland water. Red dotted line indicates Ct value limit of detection. Boxes, centerlines, and whiskers indicate the interquartile range, median, and 5th and 95th percentiles, respectively.

Test 1’s percent recoveries varied between treatments, with slow-speed DEUF using NaPP as the most variable (Figure 4, Table S5) [30]. Recoveries were statistically similar between filtering methods (TFUF; DEUF) when using either elution method (NaPP or DMEM elution) and when using fast or slow pump speeds (*p* > 0.05; n = 12–19) [30]. A Ct value < 40 was only achieved with one out of six PEG samples, with lower recovery than the other treatments. Consistent rRT-PCR results were achieved with other concentration methods; therefore, PEG was not used as a secondary concentration method moving forward with method development. After reviewing the results from Test 1, the following methods were chosen to be included in Test 2 using a natural matrix of wetland water: TFUF, NaPP (ID 1A/1B; largest recovery); DEUF, NaPP (1G/1H); DEUF, DMEM (1I/1J). Due to the inconsistent recoveries between elution methods using DEUF and slow and fast pump speeds, we decided to test a ”medium” speed for DEUF in Test 2.

### 3.2. Test 2

The three treatments that met requirements from Test 1 were tested again in Test 2 using an expanded titer series in triplicate and using a natural matrix (i.e., wetland water). The purpose of Test 2 was to determine (1) the minimum concentration for the recovery of infectious AIV and (2) whether biotic components affected the recovery of infectious AIV.

Test 2 rRT-PCR Ct values were statistically greater, corresponding to statistically lower recoveries (*p* < 0.05) than Test 1 (Figure 4; Tables S5 and S6) [30]. The raw wetland sample Ct values were more inconsistent than the autoclaved wetland, with Ct values < 40 in one or two out of three samples (Table S6) [30]. Recoveries were also smaller in the raw wetland water samples (Figure 4).

Even at low titers (10^−1^), AIV was isolated; however, the samples did not necessarily have a corresponding positive Ct value (Table S6) [26,30]. rRT-PCR percent recoveries of samples blocked or eluted using NaPP and filtered using TFUF or DEUF were statistically similar (*p* < 0.05; n = 17–32; Table S6) [30]; however, all Ct values at the spike using 10^−1^ EID_50_/mL and several spikes at the 10^0^ EID_50_/mL titer were below detection. rRT-PCR percent recoveries using DEUF and NaPP elution were greater than when using DMEM (Figure 4; Table S6) [30]. Endpoint titration percent recoveries were calculated for 12 samples in the experiment and were not statistically different (*p* > 0.05) from the rRT-PCR percent recoveries (Table S6) [30].

### 3.3. Quality Assurance/Quality Control

All six negative controls (Test 1 = 3; Test 2 = 3) were negative for AIV via rRT-PCR and VI. Neither the filtering nor elution method affected the negative control results. Changes in the DI matrix (e.g., the addition of buffer or the addition of dust; Test 1) or changes in the natural water matrix (e.g., raw water or autoclaved water; Test 2) did not affect the negative control results.

## 4. Discussion

In this study, a large-volume concentration method was developed to detect infectious AIV in waterbodies. Avian influenza viruses in environmental waters occur at low levels, requiring concentration methods to detect their presence [13,14,15]. Previous aqueous methods for detecting viruses have either used RT-PCR or smaller volumes of water (e.g., 1 L) [3,45]; however, our method was developed to be more representative of the exposome of waterfowl and other aquatic organisms utilizing the waterbodies [46]. While United States Environmental Protection Agency Method 1615 is considered a standard method for viral analysis, it was developed for non-enveloped viruses such as enterovirus and norovirus [47]. Our method development, however, focused on the concentration of the avian influenza virus (an enveloped virus) without inactivation, providing critical information on the infectivity of the virus detected in the environment and the corresponding potential risk.

Test 1 used a matrix of DI water amended with buffer and sterile dust (as a surrogate for environmental particulates) spiked with 1 × 10^3^ EID_50_/mL LP H3N8. Two filtering methods (DEUF and TFUF; Figure 2), two filtering speeds (“fast” and “slow”), two blocking/elution solutions (NaPP and DMEM), and two concentration methods (centrifugation and PEG) were tested (Table 1; Figure 1, Figure 2 and Figure 3). Test 2 used a matrix of natural wetland water (autoclaved, raw) spiked with five quantities of virus (1 × 10^3^, 1 × 10^2^, 1 × 10^1^, 1 × 10^0^, 1 × 10^−1^ EID_50_/mL LP H3N8). Two filtering methods (DEUF and TFUF), two filtering speeds (“fast” and “medium”), two blocking/elution solutions (NaPP and DMEM), and one concentration method (centrifugation) were tested (Table 1; Figure 1, Figure 2 and Figure 3). DEUF and TFUF are effective in collecting large-volume water samples for the recovery of microorganisms [13,15,18,26,48,49,50,51]. When considering methods to test, we selected solutions that were non-toxic, used in other viral recovery studies [21,52,53,54], and readily available. Several considerations informed concentration methods including ease of laboratory adaptability, biosafety concerns with aerosolization potential, time involved, and viral recovery.

The optimal protocol was dead-end ultrafiltration coupled with salt solution elution and centrifugation concentration. This method is sensitive enough to detect infectious AIV in quantities of at least 1 × 10^−1^ EID_50_/mL of wetland water and likely lower, as we did not reach a limit of detection during Test 2 (Table S6) [30]. The method outlined in this study, including use in the field and laboratory, has been validated previously using environmental samples. Infectious AIV, including the HPAI virus, was recovered from wetlands in Iowa in April 2022, providing evidence that surface water is a plausible medium for the environmental transmission of AIVs [26]. LP AIV surveillance data from these Iowa wetlands showed concentration ranges of 10^1.4^–10^2.63^ EID_50_/mL, two to three orders of magnitude larger than our tested infectious AIV detection sensitivity.

The various techniques used in this study can be compared, as the same extraction and rRT-PCR procedures were used for both Tests 1 and 2. Using a matrix of DI water with buffer and sterile dust (limiting abiotic or biotic factors; Test 1), recoveries were statistically similar between filtering methods, pump speeds, elution solutions, and concentration methods, with the exception of PEG (Figure 4) [30]. When an abiotic matrix (wetland water; Test 2) was introduced, NaPP elution was consistently greater than DMEM using DEUF (Figure 4) [30]. TFUF using NaPP elution performed similarly to DEUF using NaPP elution; however, there is a tradeoff between AIV recoveries and the rate of sample collection [48]. TFUF is substantially more complicated and labor-intensive for collecting environmental samples [15], and in our study, it did not yield statistically improved recovery (Figure 4) [30]. Therefore, the optimal technique combination for field deployment and validation for our study was DEUF with a NaPP elution solution and centrifugation concentration, as recoveries using NaPP were greater than DMEM.

Test 2 Ct values were greater and recoveries smaller than those for Test 1, likely due to the use of natural wetland water, which introduces a variety of possible inhibitors [26]. Test 1 used DI infused with sterile dust, which introduces particulates but no biotic or organic inhibitors that would accompany natural wetland water. Molecular recoveries were comparable to other AIV concentration method studies; Rönnqvist et al. [25] and Deboosere et al. [3] reported similar recoveries from spiked lake water (0.01 to 7.89% and 0.78 to 7.33%, respectively), and Ropeke [24] reported 0.3 to 6.4% recovery from spiked tap water.

In Test 2, Ct values > 40 were common in raw wetland samples at the highest (10^3^) titer and in the autoclaved wetland samples at the lowest titers (e.g., 10^−1^) (Table S6) [30]. This indicates that both biotic and abiotic factors can inhibit rRT-PCR. This introduction of inhibitors and the subsequent effects on Ct values (>40) has been noted in environmental AIV work; however, no test of inhibition was included for Test 1 or 2, as this is not included in the published molecular methods used in AIV diagnostics [3,26,45]. The introduction of inhibitors likely caused increased rRT-PCR Ct values and, therefore, the percent recoveries were likely artificially low (Figure 4, Table S6) [30]. With improved extraction and rRT-PCR techniques to aid in removing/overcoming inhibition, environmental molecular detection could be improved in future studies.

For some treatments, AIV was not detected via rRT-PCR but was positive via VI. It is possible that as little as one viral particle could result in a positive VI but be below the rRT-PCR limit of detection for that assay. Further, up to 1 mL of eluate was used in the inoculation of multiple ECEs per sample, while viral RNAs were only extracted from a 50 µL volume. The eluate was viscous and may not have allowed for a well-mixed concentrated sample. Therefore, the distribution of viral particles may not be homogenous throughout the eluate and, as such, may have led to discordant rRT-PCR and VI results.

The detection of infectious AIV in the environment is important for determining potentially transmissible viruses via waterbodies, which has implications for wildlife, poultry, and human health. This method will not only aid in the environmental surveillance of the avian influenza virus but may be relevant to the detection of other enveloped viruses in the environment.

## Figures and Tables

**Table 1:** Test 1 and Test 2 experiment information.

**TEST 1**										
**ID**	**Matrix**	**Matrix Treatment**	**Virus Titer ^¥^**	**Filtering Speed**	**Filtering Type**	**Blocking Solution**	**Elution Solution**	**Secondary Concentration**	**Tertiary** **Concentration**	**Replicate**
1A	DI water	buffer+dust	10^3^	slow	TFUF	NaPP	Sterile DI	centrifuge	centricons	n = 4
1B	DI water	buffer+dust	10^3^	fast	TFUF	NaPP	Sterile DI	centrifuge	centricons	n = 3
1C	DI water	buffer+dust	10^3^	slow	TFUF	NaPP	DMEM	centrifuge	centricons	n = 3
1D	DI water	buffer+dust	10^3^	fast	TFUF	NaPP	DMEM	centrifuge	centricons	n = 3
1E	DI water	buffer+dust	10^3^	slow	TFUF	NaPP	Sterile DI	PEG	centricons	n = 3
1F	DI water	buffer+dust	10^3^	fast	TFUF	NaPP	Sterile DI	PEG	centricons	n = 3
1G	DI water	buffer+dust	10^3^	slow	DEUF	NA	NaPP	centrifuge	centricons	n = 4
1H	DI water	buffer+dust	10^3^	fast	DEUF	NA	NaPP	centrifuge	centricons	n = 3
1I	DI water	buffer+dust	10^3^	slow	DEUF	NA	DMEM	centrifuge	centricons	n = 3
1J	DI water	buffer+dust	10^3^	fast	DEUF	NA	DMEM	centrifuge	centricons	n = 3
1M	DI water	buffer+dust	NA	fast	TFUF	NaPP	Sterile DI	centrifuge	centricons	n = 1
1N	DI water	buffer+dust	NA	fast	DEUF	NA	NaPP	centrifuge	centricons	n = 1
1O	DI water	buffer	NA	fast	DEUF	NA	DMEM	centrifuge	centricons	n = 1
**TEST 2**										
**ID**	**Matrix**	**Matrix Treatment**	**Virus Titer ^¥^**	**Filtering Speed**	**Filtering Type**	**Blocking Solution**	**Elution Solution**	**Secondary Concentration**	**Tertiary** **Concentration**	**Replicate**
2A	Wetland water	NA	10^3^	fast	TFUF	NaPP	Sterile DI	centrifuge	centricons	n = 3
2B	Wetland water	autoclave	10^3^	fast	TFUF	NaPP	Sterile DI	centrifuge	centricons	n = 3
2C	Wetland water	autoclave	10^2^	fast	TFUF	NaPP	Sterile DI	centrifuge	centricons	n = 3
2D	Wetland water	autoclave	10^1^	fast	TFUF	NaPP	Sterile DI	centrifuge	centricons	n = 2
2E	Wetland water	autoclave	10^0^	fast	TFUF	NaPP	Sterile DI	centrifuge	centricons	n = 3
2F	Wetland water	autoclave	10^−1^	fast	TFUF	NaPP	Sterile DI	centrifuge	centricons	n = 3
2G	Wetland water	NA	10^3^	medium	DEUF	NA	NaPP	centrifuge	centricons	n = 3
2H	Wetland water	autoclave	10^3^	medium	DEUF	NA	NaPP	centrifuge	centricons	n = 3
2I	Wetland water	autoclave	10^2^	medium	DEUF	NA	NaPP	centrifuge	centricons	n = 2
2J	Wetland water	autoclave	10^1^	medium	DEUF	NA	NaPP	centrifuge	centricons	n = 2
2K	Wetland water	autoclave	10^0^	medium	DEUF	NA	NaPP	centrifuge	centricons	n = 3
2L	Wetland water	autoclave	10^−1^	medium	DEUF	NA	NaPP	centrifuge	centricons	n = 3
2M	Wetland water	NA	10^3^	medium	DEUF	NA	DMEM	centrifuge	centricons	n = 3
2N	Wetland water	autoclave	10^3^	medium	DEUF	NA	DMEM	centrifuge	centricons	n = 3
2O	Wetland water	autoclave	10^2^	medium	DEUF	NA	DMEM	centrifuge	centricons	n = 2
2P	Wetland water	autoclave	10^1^	medium	DEUF	NA	DMEM	centrifuge	centricons	n = 2
2Q	Wetland water	autoclave	10^0^	medium	DEUF	NA	DMEM	centrifuge	centricons	n = 3
2R	Wetland water	autoclave	10^−1^	medium	DEUF	NA	DMEM	centrifuge	centricons	n = 3
2S	Wetland water	NA	NA	fast	TFUF	NaPP	Sterile DI	centrifuge	centricons	n = 1
2T	Wetland water	autoclave	NA	medium	DEUF	NA	NaPP	centrifuge	centricons	n = 1
2U	Wetland water	autoclave	NA	medium	DEUF	NA	DMEM	centrifuge	centricons	n = 1

ID, identifier; DI, deionized water; TFUF, tangential flow filtration; NaPP, sodium hexametaphosphate; DMEM, Dulbecco’s Modified Eagle Medium; NA, not applicable. ^¥^ egg infective dose (EID)_50_/mL.

## Data Availability

The data presented in this study are available as a USGS data release [24] [Hubbard et al. (2024)].

## Supplementary Materials

The following supporting information can be downloaded at https://www.mdpi.com/article/10.3390/v16121898/s1: Table S1: Additional solution information; Table S2: Source wetland water field parameter information for both autoclaved and raw water used as a matrix in Test 2; Table S3: National Weather Service Online Weather Data (NOWData) for stations along the transportation path of raw wetland water; Table S4: Five-minute-interval air temperature data during storage of raw wetland water before filtration in Lansing, MI; Table S5: Test 1 results; Table S6: Test 2 results; Table S7: Filter test results; Table S8: Example standard curve used to calculate rRT-PCR recovery.
